# Ternary pentagonal BXN (X = C, Si, Ge, and Sn) sheets with high piezoelectricity†

**DOI:** 10.1039/d2ra08342f

**Published:** 2023-03-24

**Authors:** Thanasee Thanasarnsurapong, Panyalak Detrattanawichai, Klichchupong Dabsamut, Intuon Chatratin, Jiraroj T-Thienprasert, Sirichok Jungthawan, Adisak Boonchun

**Affiliations:** a Department of Physics, Faculty of Science, Kasetsart University Chatuchak Bangkok 10900 Thailand adisak.bo@ku.th; b Department of Materials Science and Engineering, University of Delaware Newark Delaware 19716 USA; c School of Physics, Institute of Science, and Center of Excellence in Advanced Functional Materials, Suranaree University of Technology Muang Nakhon Ratchasima 30000 Thailand

## Abstract

The discovery of new and stable two-dimensional pentagonal materials with piezoelectric properties is essential for technological advancement. Inspired by recently reported piezoelectric materials *penta*-BCN and *penta*-BSiN, we proposed *penta*-BGeN and *penta*-BSnN as new members of the *penta*-family based on first-principles calculations. Comprehensive analyses indicated that both *penta*-BGeN and *penta*-BSnN are thermodynamically, dynamically, mechanically, and thermally stable. In terms of mechanical stability, the elastic constant decreased as lower elements in group 4A of the periodic table were used. Therefore, *penta*-BGeN and *penta*-BSnN are softer than *penta*-BCN and *penta*-BSiN. In terms of piezoelectric properties, piezoelectric stress and strain tensors increase following the same pattern. In group 4A, *penta*-BSnN had the highest intrinsic piezoelectricity, especially the *e*_22_ piezoelectric stress. Typically, the piezoelectric strain *d*_*ij*_ coefficient increases with material softness; *penta*-BSnN possessed the highest *d*_*ij*_. Thus, due to its inherent piezoelectricity, *penta*-BSnN has tremendous potential as a nanoscale piezoelectric material.

## Abstract 2

The piezoelectricity of two-dimensional pentagonal BXN (X = C, Si, Ge, and Sn) nanosheets.

## Graphical abstract

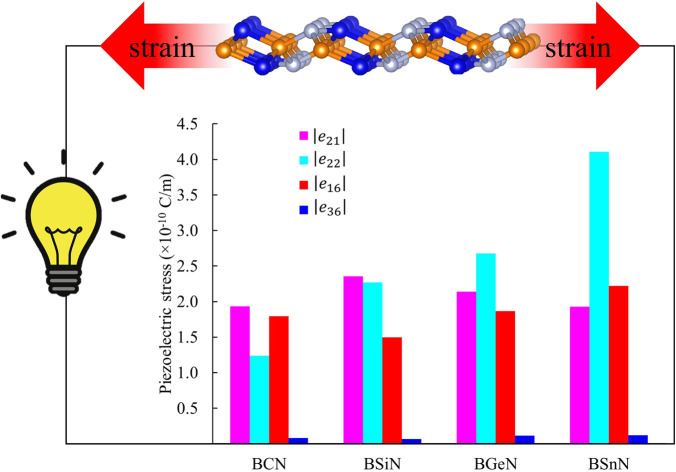

## 1 Introduction

Since the discovery of hexagonal graphene, the theoretical exploration and production of two-dimensional (2D) materials have advanced rapidly, even beyond the conventional honeycomb-like structure. A new pentagonal structure, *penta*-graphene, that resembles the Cairo pentagonal tiling proposed by Zhang *et al.*,^1^ has been extensively investigated through first-principles calculations, although it cannot yet be experimentally synthesized. Its wide band gap of 3.25 eV and high strength could lead to potential applications in nanoelectronics and nanomechanical devices. Other prominent examples are SiC_2_, CN_2_, BN_2_, B_2_C, PdS_2_, AlN_2_, PtN_2_, PdN_2_, and NiS_2_.^2–9^

In addition to binary compounds, ternary compounds offer a higher degree of freedom for tuning their electronic and structural properties. The most prominent examples are *penta*-BCN and hydrogenated *penta*-BCN,^10,11^ which possesses a strong piezoelectric response and is thermodynamically, mechanically, and thermally stable according to density functional theory (DFT). Two dimensional piezoelectric materials have the ability to convert electrical energy into mechanical energy and can find utility in a variety of applications, including sensors, flexible electronics, energy harvesting, and energy storage.^12,13^ Subsequently, *penta*-PdPSe and *penta*-PdPS were experimentally synthesized.^14^ However, *penta*-PdPSe is non-piezoelectric materials. However, in recent research, the incorporating sulfur into its surface can induce piezoelectricity from non-piezoelectricity in *penta*-PdPSe.^15^

Here, we deployed first-principles calculations to search for a novel member of the ternary pentagonal sheet family with piezoelectric properties. Inspired by the piezoelectricity of *penta*-BCN, we proposed *penta*-BGeN and *penta*-BSnN as two new members of the *penta*-2D sheet family. We found that both are thermodynamically, dynamically, mechanically, and thermally stable. *Penta*-BSnN manifests the high piezoelectricity because of its noncentrosymmetric and semiconducting characteristics. Therefore, *penta*-BSnN is a promising candidate for future nanoscale piezoelectric materials, especially for electromechanical devices.

## 2 Computational method

First-principles calculations using the framework of DFT are employed with the generalized gradient approximation (GGA) by Perdew–Burke–Ernzerhof (PBE)^16^ with the plane-wave basis projector augmented wave (PAW)^17^ method, as defined in the Vienna *Ab initio* Simulation Package (VASP).^18,19^ A kinetic energy cutoff of 500 eV is used for the plane-wave expansion. The structures are optimized until the force on each atom is less than 0.01 eV Å^−1^, and the energy convergence is set to 10^−5^ eV. The Monkhorst–Pack^20^ scheme with 9 × 9 × 1 is employed for the first Brillouin zone integration. A vacuum layer of 20 Å is added along the direction perpendicular to the layer to prevent interactions between a crystal layer and its neighboring image. To verify the dynamic stability, the PHONOPY package^21^ with an energy cutoff of 500 eV and a single *k*-point at *Γ* are used to calculate the phonon spectra over a 5 × 5 × 1 supercell. To confirm the thermodynamic stability, *ab initio* molecular dynamics (AIMD) simulations were deployed under the constant volume and temperature (NVT) ensemble, where the temperature (*T*) was controlled using the Nose–Hoover thermostat.^22–25^ The Heyd–Scuseria–Ernzerhof (HSE06)^26,27^ hybrid functional is deployed to calculate the electronic band structure because the standard approximation severely underestimates the band gaps.

## 3 Results and discussion

**Fig. 1:**
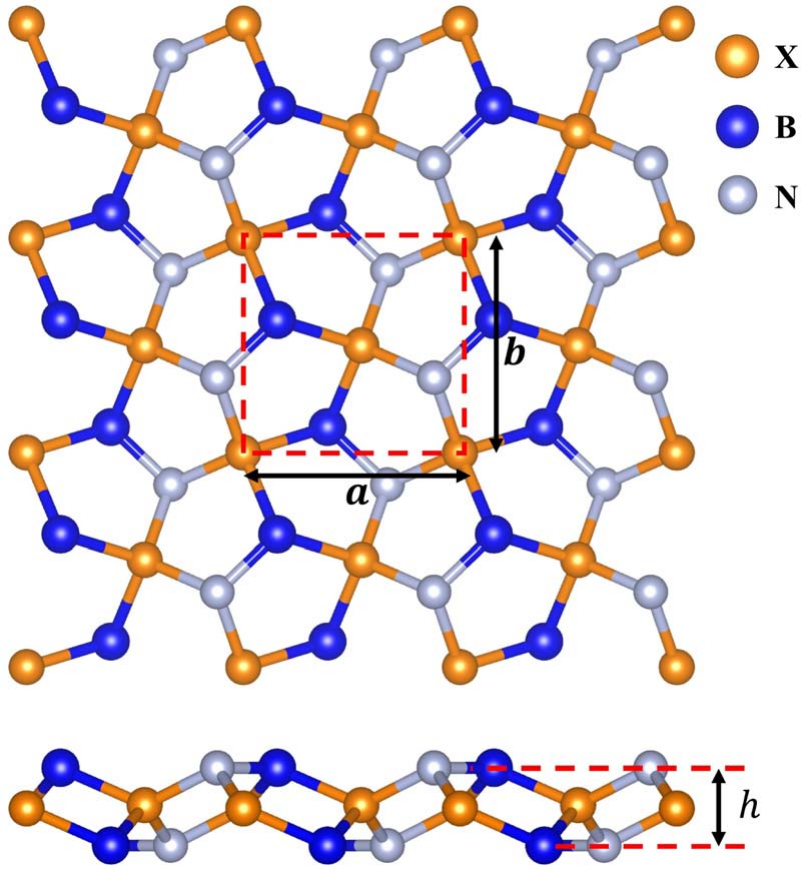
3 × 3 supercell of pentagonal BXN (X = C, Si, Ge and Sn) geometrical structures.

We first constructed *penta*-BXN (X = C, Si, Ge, and Sn) based on the reported atomic configuration of BCN in ref. 10 by replacing the C atoms with Si, Ge, or Sn atoms, as shown in Fig. 1. The pentagonal primitive unit cell, represented by the square in Fig. 1, consists of 6 atoms, including 2 B atoms, 2 X (X = C, Si, Ge, or Sn) atoms, and 2 N atoms. The calculated lattice constants (*a* and *b*) and buckling height of *penta*-BXN (X = C, Si, Ge, and Sn) are listed in Table 1. Our calculated lattice constants for *penta*-BCN and *penta*-BSiN matched previous studies.^10,28^ The lattice constants *a* and *b* increased as we go down a group in the periodic table because the atomic radii increase with elements further down a group. The buckling height *h* also increases the further down a group an element is.

**Table 1:** The calculated lattice constants *a* and *b* in Å, bucking height in Å, and the band gaps *E*^PBE^_g_ and *E*^HSE^_g_ in eV

	*a*	*b*	*h*	*E* ^PBE^_g_	*E* ^HSE^_g_
*penta*-BCN	3.670	3.631	1.314	1.70	2.91
*penta*-BSiN	4.438	4.401	1.602	1.48	2.33
*penta*-BGeN	4.607	4.590	1.640	1.59	2.42
*penta*-BSnN	5.045	5.059	1.700	1.05	1.73

In binding stability, the cohesive energy per atom was calculated, given by the following equation;^29,30^1
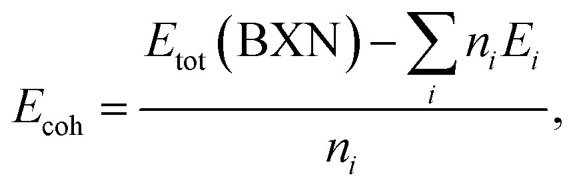
where *E*_tot_(BXN) is the total energy of the *penta*-BXN (X = C, Si, Ge and Sn); *n*_*i*_ is the number of Ni, P, and S atoms in the unit cell; and *E*_*i*_ represents the total energies of an isolated single B, X (X = C, Si, Ge or Sn), and N atoms. Our calculated cohesive energies of the *penta*-BXN (X = C, Si, Ge and Sn) are −7.69, −6.83, −6.24 and −5.84 eV per atom for *penta*-BCN, *penta*-BSiN, *penta*-BGeN and *penta*-BSnN, respectively. The negative values indicate that all *penta*-BXN are more energetically favorable than the isolated atoms. We can see that *penta*-BCN has the lowest cohesive energy per atom. It means that *penta*-BCN has a strongest binding interaction among *penta*-BXN. In comparison, the previous report cohesive energy of *penta*-graphene is −6.84 eV per atom.^31^ For the binary *penta*-carbides XC_2_, the cohesive energy of *penta*-SiC_2_, *penta*-GeC_2_, and *penta*-SnC_2_ are −5.29, −4.69, and −3.83 eV per atom, respectively.^32^

**Fig. 2:**
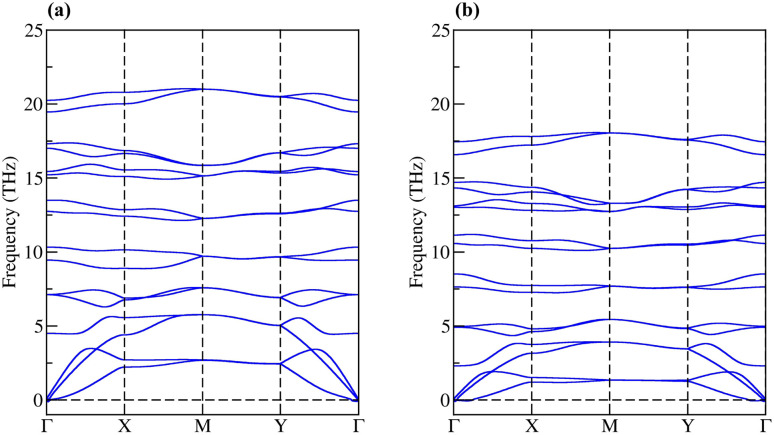
Calculated phonon band structures of (a) *penta*-BGeN and (b) *penta*-BSnN.

The dynamic stability of *penta*-BCN and *penta*-BSiN has previously been verified by calculating their phonon dispersion.^10,28^ Herein, we verify the dynamic stability of *penta*-BGeN and *penta*-BSnN by constructing phonon band structure. The dynamic stability manifests in phonon spectra containing positive frequencies throughout the Brillouin zone. In contrast, if the spectra display imaginary frequency modes (negative values), the structures contain non-restorative forces against a displacement of atoms, indicating dynamic instability. Fig. 2(a) and (b) show the calculated phonon dispersions of *penta*-BGeN and *penta*-BSnN, respectively. Thus, as shown in Fig. 2(a) and (b), there are no imaginary modes in the Brillouin zone. These spectra confirm that *penta*-BXN (X = C, Si, Ge, and Sn) are dynamically stable. We note that the imperceptible negative frequencies at *Γ*, which are only visible when the spectra are magnified, are a well-known computational error that can be effectively eliminated using the local-density approximations (LDA) functional. These negative frequencies of the phonon dispersion have also been observed in *penta*-PdSe_2_.^33^

Mechanical stability is an important property for strain engineering. Such stability can be verified from the linear elastic constants, *C*_11_, *C*_22_, *C*_12_, and *C*_66_, which are elements in the stiffness tensor. Here, the elastic constants are obtained using the finite difference method. The calculated elastic constants of *penta*-BXN (X = C, Si, Ge, and Sn) are listed in Table 2. Our calculated elastic constants were comparable to those previously calculated for *penta*-BCN and *penta*-BSiN,^10,28^ which are listed in Table 2. Typically, structures are considered mechanically stable when the linear elastic constants satisfy the conditions *C*_11_*C*_22_ – *C*_12_^2^ > 0 and *C*_66_ > 0. Notably, these conditions were met for all pentagonal BXN, confirming the mechanical stability of *penta*-BXN (X = C, Si, Ge, and Sn). In comparison, the elastic constant *C*_*ij*_ decreased with increasing atomic radii (*i.e.*, descending a group in the periodic table).

**Table 2:** The elastic constants *C*_*jk*_ (Nm^−1^), Poisson's ratio in [100] (*ν*_*a*_) and [010] (*ν*_*b*_) directions and Young's modulus (Nm^−1^) in [100] (*E*_*a*_) and [010] (*E*_*b*_) directions

	Previous work		This work			
	BCN^10^	BSiN^28^	BCN	BSiN	BGeN	BSnN
*C*_11_	223.56	114.46	222.31	116.97	100.59	66.82
*C*_12_	4.90	12.76	4.03	13.06	10.69	17.85
*C*_22_	187.39	111.21	187.39	112.60	98.45	66.64
*C*_66_	105.38	48.97	105.38	49.36	47.09	32.57
*ν*_*a*_	0.022	0.11	0.018	0.112	0.106	0.267
*ν*_*b*_	0.026	0.11	0.022	0.116	0.109	0.268
*E*_*a*_	223.45	113	222.24	115.51	99.46	62.05
*E*_*b*_	189.03	109	187.30	111.08	97.30	61.86

Next, we evaluated the 2D Young's modulus in the [100] and [010] directions (*i.e.*, along the plane), which is obtained by *E*_*a*_ =(*C*_11_^2^ − *C*_12_^2^)/*C*_11_ and *E*_*b*_ = (*C*_22_^2^ − *C*_12_^2^)/*C*_22_. Poisson's ratios in the [100] and [010] directions are *ν*_*a*_ = *C*_12_/*C*_11_ and *ν*_*b*_ = *C*_12_/*C*_22_, respectively. Young's modulus and Poisson's ratio for all pentagonal BXNs are listed in Table 2. The values of *penta*-BCN and *penta*-BSiN are close to those previously reported, *E*_*a*_ = 223.45 Nm^−1^ and *E*_*b*_ = 189.03 Nm^−1^ for *penta*-BCN, and *E*_*a*_ = 113 Nm^−1^ and *E*_*b*_ = 109 Nm^−1^ for *penta*-BSiN. The Young's modulus decreased when we went down a group in the periodic table, while Poisson's ratio increased. These indicate that the pentagonal materials softened as larger elements in group 4A were chosen, and the materials were likely to deform in directions perpendicular to [100] and [010]. Similarly, the elastic constants and Young's modulus of *penta*-XC_2_ (X = C, Si, Ge, and Sn) have been reported.^32^ The calculated Young's modulus values for *penta*-XC_2_ (X = C, Si, Ge, and Sn) are 266.22, 138.54, 121.65, and 81.23 Nm^−1^, respectively. It can be observed that in *penta*-XC_2_ (X = C, Si, Ge, and Sn), the Young's modulus decreases as we move down the group in the periodic table. This behavior is similar to that of *penta*-BXN, where larger elements tend to soften the material. The near-zero Poisson's ratios of *penta*-BCN indicate that *penta*-BCN maintains its dimensions under compression and extension along the in-plane directions.

**Fig. 3:**
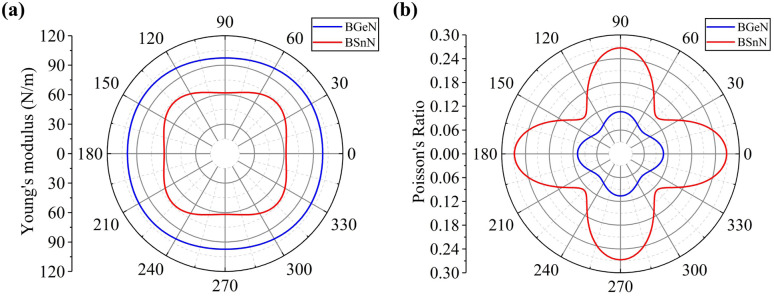
Variation of (a) in-plane Young's modulus, and (b) Poisson's ratio for *penta*-BGeN and *penta*-BSnN. Note that solid blue and red lines represent *penta*-BGeN and *penta*-BSnN, respectively.

We used eqn (1) and (2) to calculate Young's modulus (*E*) and Poisson's ratio (*ν*) along an arbitrary angle *θ*,^34–36^ which is the angle relative to the *x* direction, in order to further investigate how the mechanical properties of *penta*-BXN depend on its crystal orientation.2
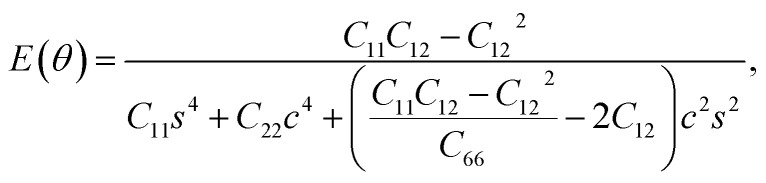
3
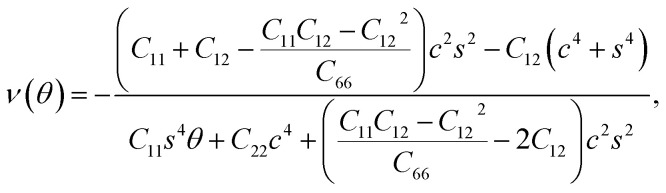
where *c* = cos *θ* and *s* = sin *θ*. The calculated results for *penta*-BGeN and *penta*-BSnN are plotted in Fig. 3, while the calculated results for *penta*-BCN and *penta*-BSiN have been provided previously.^10,28^ The maxima of the in-plane Young's modulus in *penta*-BGeN and *penta*-BSnN are located around the [110] direction (40 for *penta*-BGeN and 45 for *penta*-BSnN). Meanwhile, the minima in-plane Young's modulus of both are along [010] (90). In *penta*-BGeN, the minimum and maximum Poisson's ratios are found along [110] (43) and [010] (90), respectively. In *penta*-BSnN, the minimum and maximum Poisson's ratios are located at the same angle as *penta*-BGeN but have higher positive values. Because the in-plane Young's modulus and Poisson's ratios depend on crystal orientation, *penta*-BXN (X = C, Si, Ge, and Sn) exhibit anisotropic mechanical properties that can be explained by their lattice constants, where *a* ≠ *b*.

Using mapping of indices, the relationship among the elastic (*C*_*jk*_), piezoelectric stress (*e*_*ik*_), and strain tensors (*d*_*ij*_) is

4 *e*_*ik*_ = *d*_*ij*_*C*_*jk*_

Specifically, for a pentagonal system, the relationship between the three variables becomes5
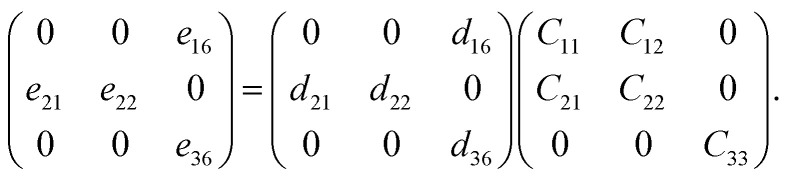


Piezoelectric tensors *e*_*ik*_ are calculated using density functional perturbation theory (DFPT) as implemented in the VASP package. The elastic stiffness tensors are calculated by using the finite difference method mentioned before. Therefore, the elements of the strain tensors, *d*_16_, *d*_21_, *d*_22_ and *d*_36_, are derived:6
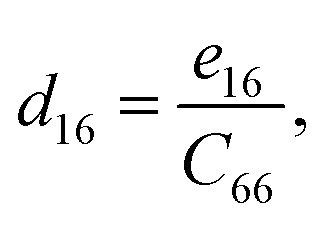
7
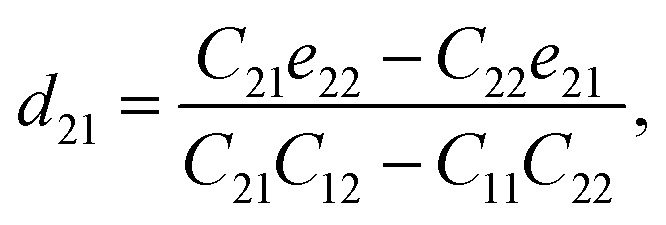
8
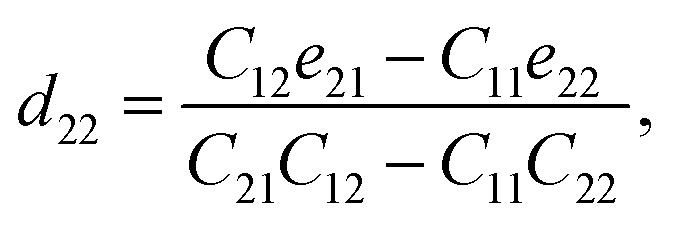
9
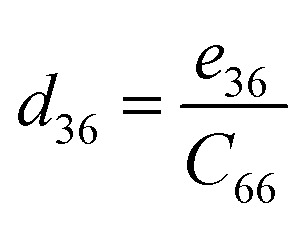


According to eqn (5)–(8), the piezoelectric strain tensors *d*_*ij*_, which are listed in Table 3, are derived from *C*_*jk*_, as listed in Table 2, and *e*_*ik*_, as listed in Table 3. Our calculated piezoelectric strain tensors, *d*_*ij*_, and piezoelectric stress tensors, *e*_*ik*_, were comparable to those of previous work in *penta*-BCN and *penta*-BSiN,^10,28^ which are also listed in Table 3. Note that the piezoelectric stress tensors *e*_21_ and *e*_22_ refer to the induced polarization along the *x*- and *y*-directions, respectively, when the strain is applied along the *y*-direction. Meanwhile, *e*_16_ and *e*_36_ represent the induced polarization along the *xy*-direction when the strain is applied along the *x*- and *y*-directions, respectively. The negative values of *e*_*ik*_ and *d*_*ij*_ refer to the opposite direction to the strain displacement. From the calculation results, the piezoelectric stress *e*_*ik*_ and strain *d*_*ij*_ tensors tended to increase as the substituted atoms went down the group in the periodic table, which is illustrated in ESI Fig. S1.† It can be inferred that the softer materials possess larger *d*_*ij*_ coefficients. The piezoelectric strain tensor *d*_*ij*_ of *penta*-BSnN is largest because *penta*-BSnN possessed the smallest elastic constants. Typically, the piezoelectric strain *d*_*ij*_ coefficient increases with material softness.^37^ The piezoelectric stress tensor *e*_22_ of *penta*-BSnN is about 3.3 times higher than that of *penta*-BCN. In addition, all of the piezoelectric coefficients (*e*_21_, *e*_16_) of *penta*-BXN are higher than those of two dimensional *h*-BN (*e*_11_ = 1.38 × 10^−10^ C m^−1^).^38^

**Table 3:** Piezoelectric stress tensors *e*_*ik*_ in unit of × 10^−10^ C m^−1^ and piezoelectric strain tensors *d*_*ij*_ of *penta*-BXN (X = C, Si, Ge and Sn) in the unit of pm V^−1^

	Previous work		This work			
	BCN^10^	BSiN^28^	BCN	BSiN	BGeN	BSnN
*e*_21_	1.93	3.78	1.93	2.35	2.14	1.93
*e*_22_	−1.24	−2.40	−1.24	−2.27	−2.68	−4.11
*e*_16_	1.80	0.84	1.80	1.50	1.86	2.22
*e*_36_	−0.08	−0.26	−0.081	−0.068	−0.113	−0.121
*d*_21_	0.878	3.59	0.882	2.27	2.45	4.88
*d*_22_	−0.678	−2.57	−0.680	−2.28	−2.98	−7.47
*d*_16_	1.72	1.71	1.70	3.03	3.96	6.81
*d*_36_	−0.076	−0.53	−0.076	−0.139	−0.240	−0.372

**Fig. 4:**
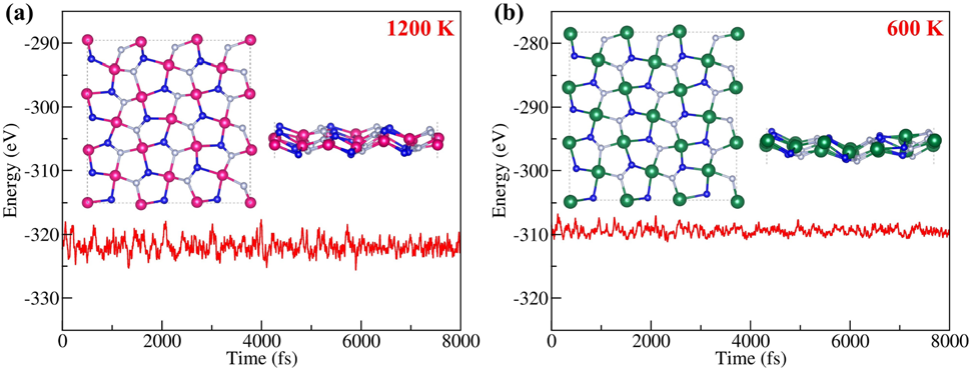
Total energy fluctuation with time during the AIMD simulation at (a) 1200 K of *penta*-BGeN and (b) 600 K of *penta*-BSnN.

The thermal stabilities of *penta*-BGeN and *penta*-BSnN were examined *via ab initio* molecular dynamic simulations. Supercells with a 3 × 3 × 1 duplication of the unit cell are used. The molecular dynamics simulations are performed for 8 ps with a time step of 1 fs at 1200 K. Fig. 4(a) shows the fluctuation of the potential energy of *penta*-BGeN throughout the simulation at 1200 K. *Penta*-BGeN is thermally stable at 1200 K. However, *penta*-BSnN is unable to stand at 1200 K, as shown in ESI Fig. S2.† Therefore, we lower the simulation temperature to 600 K. Finally, *penta*-BSnN possesses thermal stability around 600 K, as illustrated in Fig. 4(b).

**Fig. 5:**
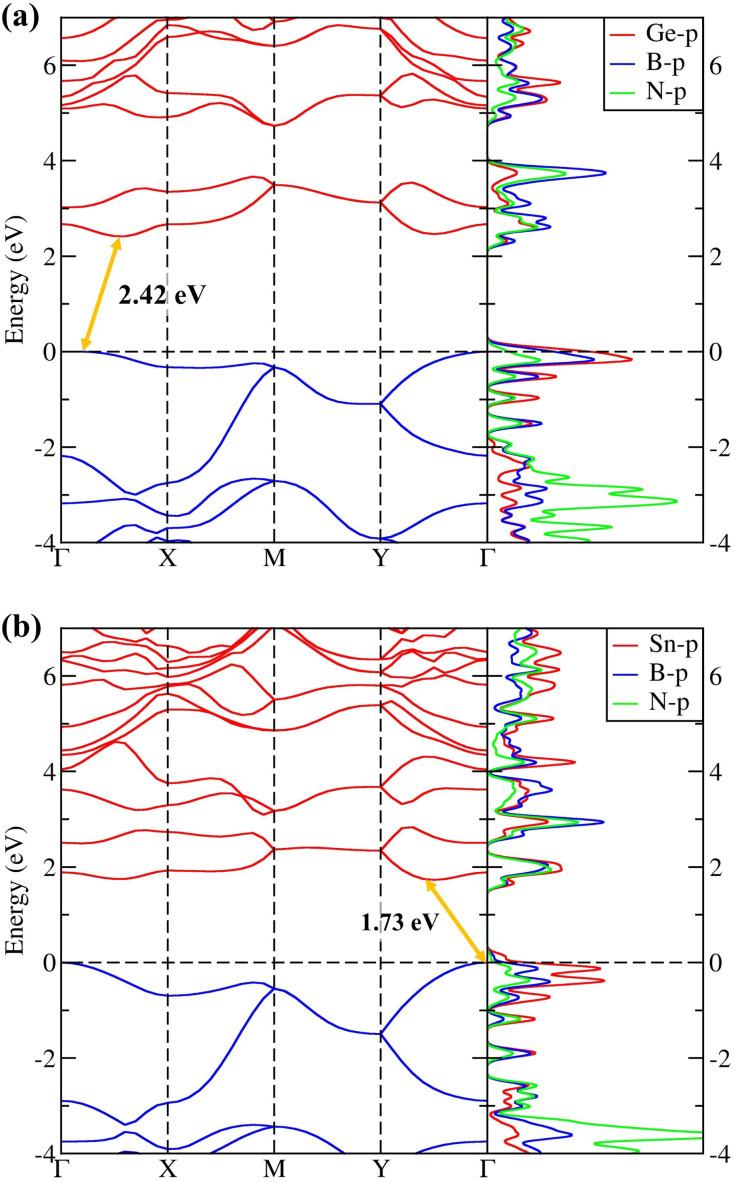
The electronic band structure and partial Density of States (pDOS) of (a) *penta*-BGeN and (b) *penta*-BSnN.

The electronic properties of *penta*-BGeN and *penta*-BSnN were first explained by the calculated Heyd–Scuseria–Ernzerhof (HSE06)^27^ band structure, as shown in Fig. 5 When the PBE functional^16^ was used, the electronic band gap showed indirect band gaps of 1.59 and 1.05 eV for *penta*-BGeN and *penta*-BSnN, as listed in Table 1. The standard DFT underestimates the band gap.^39–41^ Therefore, the HSE06 functional,^26,27^ which mixes 75% HF exchange and 25% PBE exchange, was also employed to address this issue. With the use of this functional, a larger HSE band gap of 2.42 eV was obtained for *penta*-BGeN while a larger HSE band gap of 1.73 eV was achieved for *penta*-BSnN. Both had an indirect band gap character. As shown in ESI Fig. S1,† the valence band maximum (VBM) of *penta*-BGeN and *penta*-BSnN are found at *Γ* point when HSE06 was used, while the conduction band minimum (CBM) was established along the *Γ* − *X* path for *penta*-BGeN, and the along *Γ* − *Y* path for *penta*-BSnN.

Next, we will discuss the effect of band edge position on photocatalytic water splitting. Recent density functional theory (DFT) studies have shown that penta-materials are among the possible candidates for photocatalytic water splitting.^28,32^ In water splitting, the band edges position of VBM must cover the redox/oxidation level potentials of water. The standard redox potentials with pH of oxygen evolution reaction (OER) is *E*_O_2_/H_2_O_ = −5.67 + pH × 0.059 eV, while standard redox potentials with pH of hydrogen evolution reaction (HER) can be express as *E*_H^+^/H_2__ = −4.44 + pH × 0.059 eV.^42,43^ In the present study, we present the calculated positions of the VBM and CBM with respect to the vacuum level, as shown in ESI Fig. S3.† Our results reveal that the VBM shifts rapidly with increasing pH, while the CBM remains relatively constant. Although, at a pH of 7 (as indicated by the red dashed line), the CBM band edges of all the BXN (X = C, Si, Ge, and Sn) are above the hydrogen evolution potential (H^+^/H_2_), only the *penta*-BCN material has the VBM band edges below the oxygen evolution potential (O_2_/H_2_O). These findings suggest that *penta*-BCN is a promising material for water-splitting processes, as it can catalyze both the hydrogen and oxygen evolution reactions at pH 7. Additionally, a decrease in pH (as indicated by the black dashed line) leads to a downward shift in the oxidation and reduction potentials of water, which favors the HER but is detrimental to the OER. Therefore, *penta*-BCN is the only BXN material suitable for OER under highly acidic conditions (pH = 0) in water-splitting processes.

**Fig. 6:**
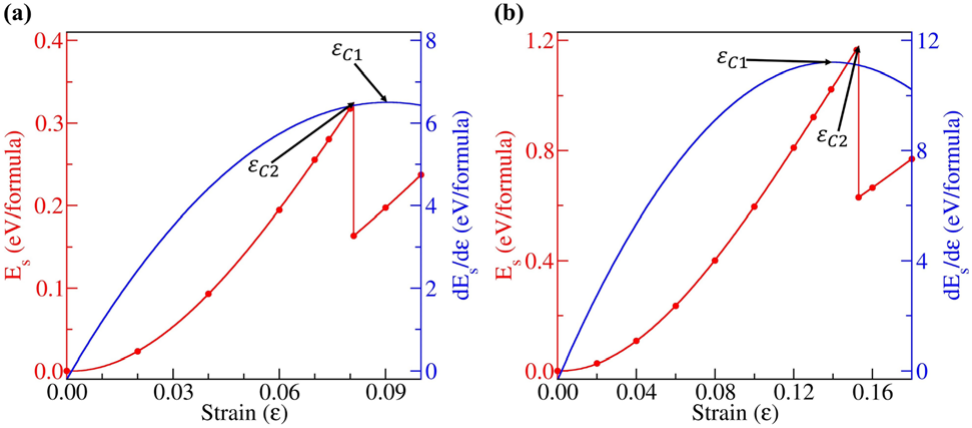
Strain–stress relations for (a) *penta*-BGeN and (b) *penta*-BSnN with biaxial strains. The vertical dashed lines indicate the critical structure instability.

The band gaps of 2D materials can potentially be modulated by strain. Thus, we also investigated how the band gaps of *penta*-BGeN and *penta*-BSnN change with additional biaxial strain from equilibrium (*i.e.*, zero strain) to the critical strain point. In order to avoid the interaction of periodic conditions, the 2 × 2 × 1 supercell has been adopted to release the special constraints. The values of two critical strains, (*ε*_*C*1_ and *ε*_*C*2_), can be inferred from Fig. 6. The first is the point where the derivative curve reaches its maximum turning point. This point is widely known as the bond-breaking point. The second is the point at which the plastic range begins to manifest irreversible deformations (reconstruction process). Above *ε*_*C*2_, the strain energy always increases, and the system maintains its reconstructive structure. In *penta*-BGeN, *ε*_*C*1_ occurred at nearly *ε* = 0.14 while it occurred at nearly *ε* = 0.08 in *penta*-BSnN. This means that *penta*-BGeN could withstand biaxial strain before mechanical failure at these critical points. However, in *penta*-BGeN, structural instability occurred at *ε*_*C*2_, at which point the structure starts to collapse.

## 4 Conclusions

By using DFT-based calculations, we theoretically explored *penta*-BGeN and *penta*-BSnN as the new members in the *penta*-2D family. Both *penta*-BGeN and *penta*-BSnN are dynamically, mechanically, and thermally stable, as comprehensively verified by the phonon dispersion, elastic constants, and molecular dynamics simulations, respectively. *Penta*-BGeN and *penta*-BSnN are semiconductors with indirect band gaps of 2.42 and 1.73 eV, respectively. *Penta*-BGeN and *penta*-BSnN are soft materials because their elastic constants decrease as we go down the group in the periodic table. The elements of the piezoelectric stress and strain tensors increase as we move down a group in the periodic table. In the same family, *penta*-BSnN yields the highest intrinsic piezoelectricity, especially the *e*_22_ piezoelectric stress. Typically, the piezoelectric strain, *d*_*ij*_, coefficient increases with material softness, yielding the greatest *d*_*ij*_ for *penta*-BSnN. Thus, *penta*-BSnN has promising prospects as a nanoscale piezoelectric material due to its inherent piezoelectricity.

## Conflicts of interest

There are no conflicts to declare.

## Supplementary Material

RA-013-D2RA08342F-s001
