# Supplementary material for: Ternary pentagonal BXN (X = C, Si, Ge, and Sn) sheets with high piezoelectricity
Source: RSC Adv. 2023 Mar 24;13(14):9636–41. doi: 10.1039/d2ra08342f (PMC10037298; doi:10.1039/d2ra08342f)
Supplement: RA-013-D2RA08342F-s001 [file RA-013-D2RA08342F-s001.pdf]

Electronic Supplementary Material (ESI) for RSC Advances.
This journal is © The Royal Society of Chemistry 2023

## Supplementary information for

# "Ternary Pentagonal BXN (X = C, Si, Ge and Sn) Sheets with High Piezoelectricity"

Thanasee Thanasarnsurapong,[†] Panyalak Detrattanawichai,[†] Klichchupong Dabsamut,[†] Intuon Chatratin,[‡] Jiraroj T-Thienprasert,[†] Sirichok Jungthawan,[¶] and Adisak Boonchun[*,†]

[†]Department of Physics, Faculty of Science, Kasetsart University, Bangkok 10900, Thailand
[‡] Department of Materials Science and Engineering, University of Delaware, Newark, DE, 19716, USA
[¶]School of Physics, Institute of Science, and Center of Excellence in Advanced Functional Materials, Suranaree University of Technology, Muang, Nakhon Ratchasima, 30000, Thailand

[*]E-mail: Adisak.bo@ku.th

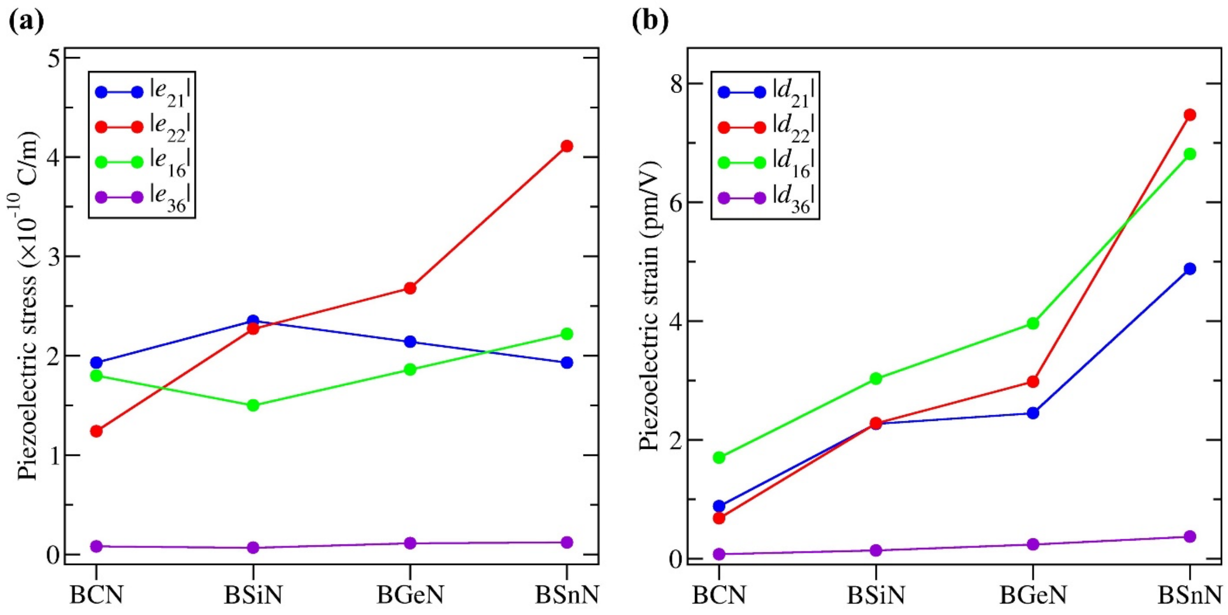


**FIG S1** The (a) piezoelectric stress tensors and (b) piezoelectric strain tensors of *penta*-BXN (X=C, Si, Ge and Sn)

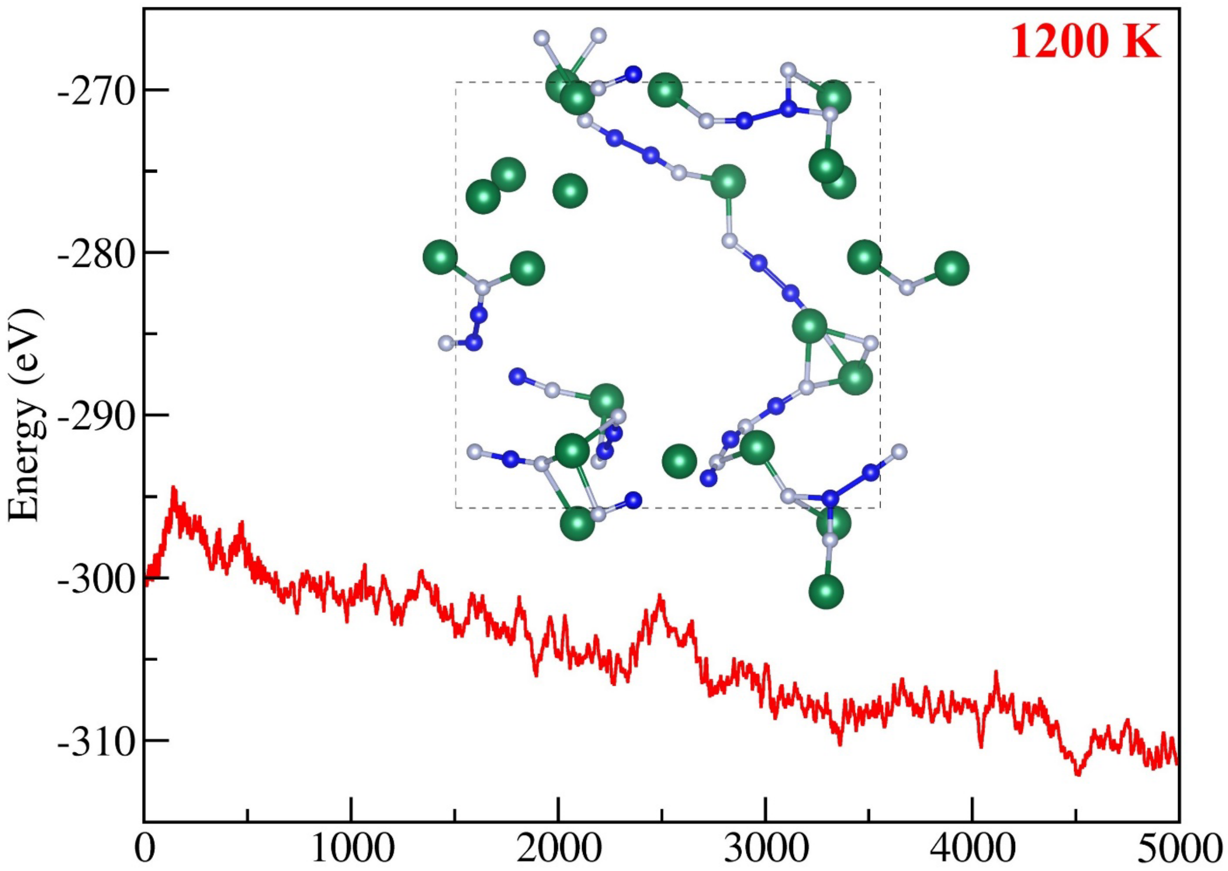


**FIG S2** MD simulation of *penta*-BSnN at temperature of 1200 K

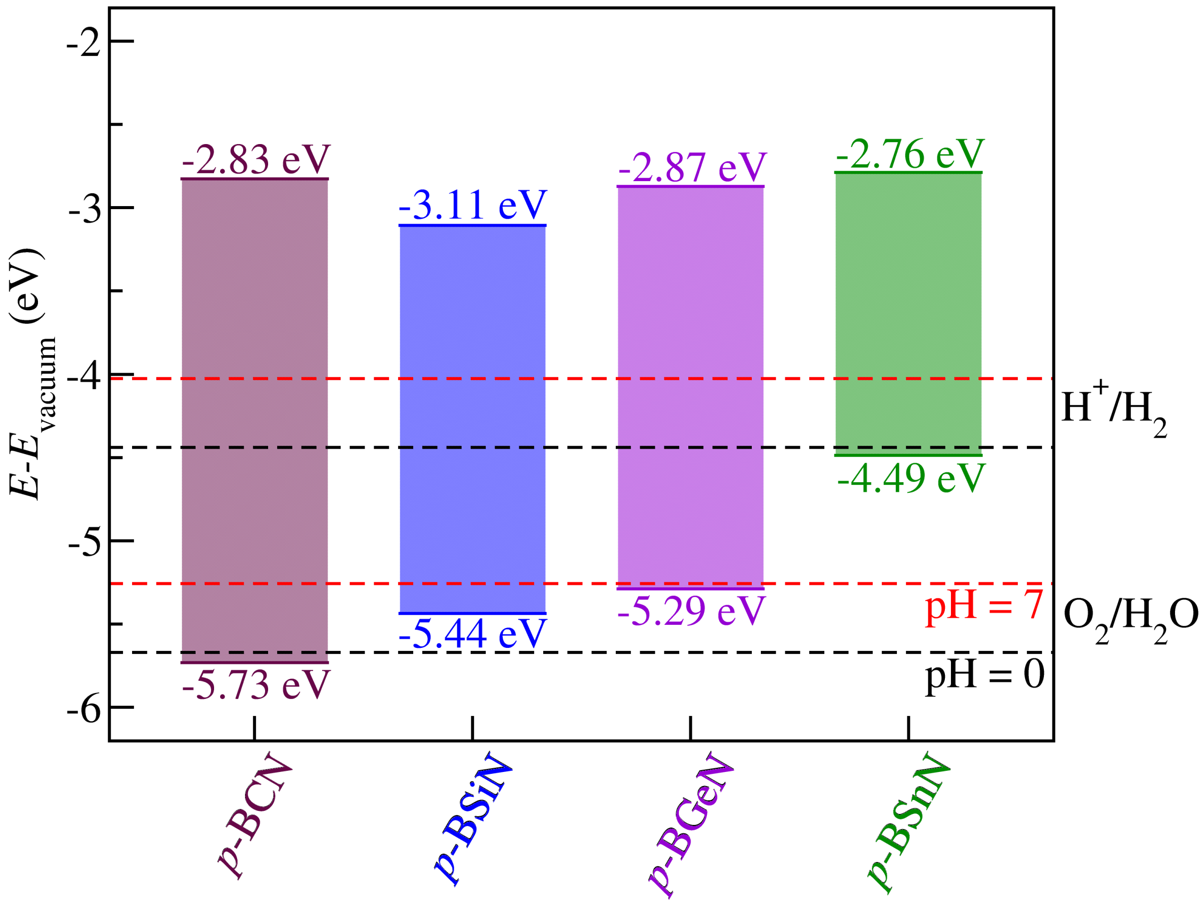


**FIG S3** Band edge alignment of the ternary *penta*-BXN with relation to the redox potentials for water-splitting at pH = 0 (black dash line) and pH = 7 (red dash line).
